# Molecular characteristics and antimicrobial resistance profiles of Carbapenem-Resistant *Klebsiella pneumoniae* isolates at a tertiary hospital in Nanning, China

**DOI:** 10.1186/s12866-023-03038-x

**Published:** 2023-10-28

**Authors:** Xianzhen Wei, Qiuxiang Li, Yu He, Linlin Li, Shan Li, Taijie Li

**Affiliations:** 1 Department of Clinical Laboratory, The First Affiliated Hospital of Guangxi Medical University, Nanning, China; 2 Department of Clinical Laboratory, Joint Logistics Support Force of the Chinese People’s Liberation Army, 923 Hospital, Nanning, China; 3 Department of Clinical Laboratory, Wuming Hospital of Guangxi Medical University, Nanning, China

**Keywords:** Carbapenem-resistant *Klebsiella pneumoniae*, Antimicrobial susceptibility, Carbapenemases, MLST

## Abstract

**Purpose:**

Carbapenem resistant *Klebsiella pneumoniae* is associated with nosocomial infections and can cause high mortality, which poses great threat to human health. This study was aimed at investigating the molecular epidemiology and antimicrobial resistance profiles of carbapenem resistant *Klebsiella pneumoniae* isolates and providing clues for management and control of carbapenem resistant *Klebsiella pneumoniae* infections.

**Methods:**

A total of 2324 *Klebsiella pneumoniae* strains were isolated from the First Affiliated Hospital of Guangxi Medical University from June 2018 to October 2020, and 103 carbapenem resistant *Klebsiella pneumoniae* strains from inpatients were collected, and the specimens mainly came from the sputum, urine, secretions, and blood. The antimicrobial susceptibility tests were performed using the VITEK 2 Compact system or the Kirby–Bauer disk-diffusion method. The resistance genes were detected by polymerase chain reaction and sequencing. The homology analysis of carbapenem resistant *Klebsiella pneumoniae* strains was performed by multilocus sequence typing.

**Results:**

Antimicrobial susceptibility results showed that the 103 carbapenem resistant *Klebsiella pneumoniae* strains were resistant to most common antibiotics. Resistance genes detection showed that the carbapenem resistant *Klebsiella pneumoniae* isolates mainly carried metallo-beta-lactamase, and the predominant gene was NDM-1. The homology analysis found that the major ST type were ST11, follow by ST15 and ST17.

**Conclusion:**

The carbapenem resistant *Klebsiella pneumoniae* isolates in our study shown resistance to most common antibiotics. Of the 103 carbapenem resistant *Klebsiella pneumoniae* strains, 91 strains (88.35%) carried carbapenemases genes, and NDM was the predominant carbapenemase gene detected. ST11 was the major ST typing of carbapenem resistant *Klebsiella pneumoniae* in our hospital. Our finding may play a role in control and management of the carbapenem resistant *Klebsiella pneumoniae* infections and guiding clinical antibiotic therapy. In addition, metallo-beta-lactamase should be served as a key target to be monitored in carbapenem resistant *Klebsiella pneumoniae* infection.

## Introduction

*Klebsiella pneumoniae* (KP) is a gram-negative encapsulated bacterium that resides in the environment, such as surface waters, soil, medical devices, human nasopharynx and gastrointestinal tract [1]. It can cause a wide range of infections, including respiratory tract infections, urinary tract infections, liver abscesses and bacteremia [2]. Carbapenems antibiotics are commonly used in severe KP infections and thought to be the last line of defense [3]. However, exposure to carbapenems, admission to ICU and longer length of hospital stay may increase the chance of carbapenem resistant *Klebsiella pneumoniae* (CRKP) infections [4, 5]. In most cases, carbapenems were prescribed when empirical antibiotics like cephalosporins, fluoroquinolone, and β-lactam/β-lactamase inhibitor combination showed resistance to KP. With carbapenems widely used, CRKP infections have increased, which limit the choice of antibiotic therapy and related to high mortality [6, 7]. The resistance mechanisms of CRKP mainly include production of carbapenemases, upregulation of efflux pump systems, lower membrane permeability and alteration of penicillin-binding proteins [8, 9]. Among them, production of carbapenemases is one of the most important mechanisms. The distribution of carbapenemases is distinct in different regions. To date, rare information about CRKP molecular epidemiology from Nanning area. Therefore, our study investigated the molecular epidemiology and antimicrobial resistance profiles of CRKP in Nanning city, aiming at providing useful clues for infection control and rational antibiotics use.

## Materials and methods

### Bacterial isolation and identification

103 non-duplicate CRKP strain were collected from the inpatients from The First Affiliated Hospital of Guangxi Medical University from June 2018 to October 2020, and informed consent was obtained from the patients before collecting samples. The main source of the specimens were sputum, urine, secretions, and blood. The specimens were collected by nurses and transported to the clinical laboratory, then inoculated on the appropriate plates based on their source. The plates were incubated at 35℃ for 18-24 h, and the positive growth organisms were further identified. All CRKP strains were identified by the VITEK2 Compact system (bioMérieux, Marcy l’Etoile, France) or matrix-assisted laser desorption/ionization time-of-flight mass spectrometry (MALDI-TOF MS) (Autof ms1000, Zhengzhou Antu Biological Engineering Co., LTD).

### Antimicrobial susceptibility testing

Antimicrobial susceptibility tests were performed using the VITEK 2 Compact system or the Kirby–Bauer disk-diffusion method, except for polymyxin B, which was performed with broth microdilution testing, and the results were interpreted as recommended by the Clinical and Laboratory Standards Institute (CLSI), version 2023 [10]. Klebsiella pneumoniae (ATCC700603), Escherichia coli (ATCC25922), and Pseudomonas aeruginosa (ATCC27853) were used as the quality control bacterial strains.

### Carbapenemases phenotype experiment

All CRKP strains were performed by Modified carbapenem inactivation test (mCIM) and EDTA modified carbapenem inactivation test (eCIM) according to the CLSI guideline [10]. The procedures for the mCIM and eCIM were as follows: A 1-µL loopful of CRKP isolate was resuspended in two tubes containing 2 mL of TSB. One tube was supplemented with 20 µL of 0.5 mol/L EDTA, while the other tube remained free from EDTA (mCIM). Next, a meropenem disk was placed in each tube, and the tubes were further incubated at 35 °C for 4 h ± 15 min. Then the disks were removed from the tubes and transferred onto MH agar plates that were freshly inoculated with a 0.5 McFarland suspension of carbapenem-susceptible E. coli ATCC25922. The plates were incubated at 35 °C for 16 to 20 h before recording the zone sizes [11, 12]. The positive result of mCIM test revealed that the strain produced carbapenemases. Both mCIM test and eCIM test result were positive revealed that the strain produced metallo-beta-lactamase (MBL). While the positive result of mCIM test and negative result of eCIM test indicated that the strain produced serine carbapenemases.

### Resistance genes detection

Carbapenemases genes including KPC, NDM, IMP, VIM, and OXA-48 were detected by PCR. The positive PCR amplicons were sequenced, and the nucleotide sequences obtained were analyzed and compared with those available in the National Center for Biotechnology Information (NCBI) GenBank database (https://blast.ncbi.nlm.nih.gov/Blast.cgi).

### Multilocus sequence typing (MLST)

MLST of CRKP was identified using seven conserved housekeeping genes (bla_gapA_, bla_infB_, bla_mdh_, bla_pgi_, bla_phoE_, bla_rpoB_ and bla_tonB_) according to the protocol available at MLST Pasteur website (https://bigsdb.pasteur.fr/klebsiella/primers-used/). The housekeeping genes sequences presented within CRKP were assigned as distinct alleles representing the allelic profile or specific ST at the loci for each isolate.

### Statistical analysis

Antimicrobial susceptibility results were analyzed using WHONET 5.6 software. Categorical variables were expressed as counts or counts/total (percentages).

## Results

### Bacterial isolation and identification

A total of 103 non-repetition CRKP strains were collected. Among the 103 CRKP infection inpatients, there were 67 males (65.05%) and 36 females (34.95%); The inpatients predominantly came from intensive care unit (ICU) ( 29.13%, 30/103), traditional Chinese medicine department (16.50%,17/103), neonatology department(13.59%,14/103), and pediatric department (9.71%,10/103). Source of the isolates mainly included sputum (43.69%,45/103), urine (25.24%, 26/103), secretions(9.71%, 10/103), and blood(6.80%, 7/103).

### Antimicrobial susceptibility testing

The 103 CRKP isolates showed high resistance to most antibiotics, especially ertapenem, cefazolin, cefuroxime, cefotaxime, ampicillin/sulbactam, amoxicillin/clavulanic acid, and ticarcillin/clavulanic acid (100%). Meanwhile, the resistance rates of meropenem, ceftazidime, cefepime, cefoxitin, cefoperazone/sulbactam, piperacillin/tazobactam were higher than 90%. In contrast, the CRKP strains showed less resistance to tigecycline and polymyxin B, with a rate of 2.91% and 6.80%, respectively. As shown in Table 1.

**Table 1:** Antimicrobial susceptibility results

Antibiotic	R (%)	I (%)	S (%)
ertapenem	103(100.00)	0(0.00)	0(0.00)
meropenem	94(91.26)	4(3.89)	5(4.85)
imipenem	91(88.35)	6(5.82)	6(5.83)
cefazolin	103(100.00)	0(0.00)	0(0.00)
cefuroxime	103(100.00)	0(0.00)	0(0.00)
cefotaxime	103(100.00)	0(0.00)	0(0.00)
ceftazidime	102(99.03)	1(0.97)	0(0.00)
cefepime	101(98.06)	2(0.94)	0(0.00)
aztreonam	91(88.35)	1(0.97)	11(10.68)
cefoxitin	102(99.03)	1(0.97)	0(0.00)
ampicillin/sulbactam	103(100.00)	0(0.00)	0(0.00)
amoxicillin/clavulanic acid	103(100.00)	0(0.00)	0(0.00)
piperacillin/tazobactam	96(93.20)	5(4.86)	2(1.94)
cefoperazone/sulbactam	102(99.03)	1(0.97)	0(0.00)
ticarcillin/ clavulanic acid	103(100.00)	0(0.00)	0(0.00)
gentamicin	52(50.49)	1(0.97)	50(48.54)
amikacin	36(34.95)	3(2.91)	64(62.14)
tobramycin	44(42.72)	10(9.71)	49(47.57)
minocycline	37(35.92)	15(14.57)	51(49.51)
levofloxacin	65(63.11)	28(27.18)	10(9.71)
ciprofloxacin	81(78.64)	11(10.68)	11(10.68)
moxifloxacin	69(66.99)	21(20.39)	13(12.62)
cotrimoxazole	68(66.02)	0(0.00)	35(33.98)
nitrofurantoin	33(32.04)	16(15.53)	54(52.43)
chloramphenicol	66(64.08)	5(4.85)	32(31.07)
tigecycline	3(2.91)	5(4.86)	95(92.23)
Polymyxin B	7(6.80)	96(93.20)	-

Note: data expressed as n (%). R, resistant; I, intermediate; S, sensitive. -: undefined

### Carbapenemases phenotype experiment and resistance genes detection

Of the 103 CRKP strains, 12 strains (11.65%) without producing carbapenemases, while 91 strains (88.35%) carried carbapenemases genes, of which 68 strains (74.73%) produced MBL, and 23 strains (25.27%) produced serinase. Among the 91 strains, 38 strains (41.76%) carried NDM-1, 25 strains (27.47%) carried KPC-2, and 25 strains (27.47%) carried NDM-5. As shown in Table 2.

**Table 2:** Resistance genes of 91 CRKP strains

Resistance gene	Strains	Percentage
NDM-1	38	41.76%
NDM-5	25	27.47%
KPC-2	25	27.47%
OXA-181	3	3.30%
IMP-4	2	2.20%
VIM	0	0.00%

### Multilocus sequence typing

**Fig. 1:**
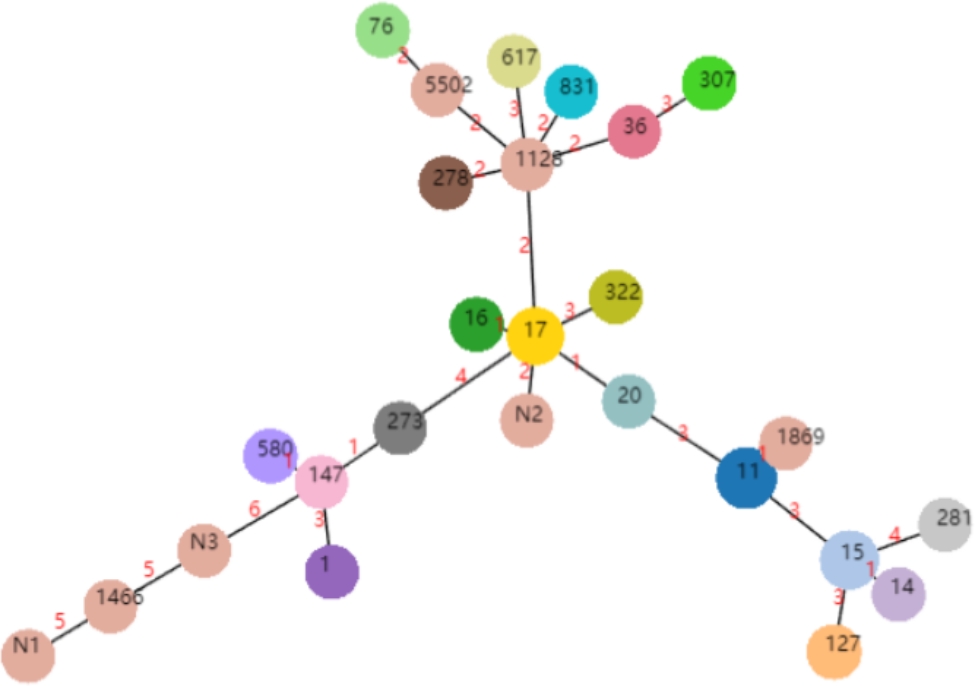
Minimal spanning tree (MST) of CRKP strains. Each circle corresponds to an ST, N1、N2、N3 represent untyped ST. The size of each circle is proportional to the number of strains. The numbers in red between two circles correspond to the numbers of allelic differences

A total of 84 CRKP strains obtained ST typing. The dominant ST typing were ST11, follow by ST15 and ST17. Among them, 21 ST11 strains (25.00%) carried KPC-2,15 ST15 strains (17.86%) carried NDM-5, and 11 ST17 strains (13.10%) carried NDM-1. As shown in Table 3. Minimal spanning tree (MST) of CRKP strains is shown in Fig. 1.

**Table 3:** ST typing and resistance genes of CRKP strains

ST	Resistance gene	Positive Strains	Percentage
ST11	KPC-2	21	25.00
ST15	NDM-5	15	17.86
ST17	NDM-1	11	13.10
ST127	NDM-5	4	4.76
ST16	OXA, unknown MBL	3	3.57
ST76	NDM-1, NDM-5	3	3.57
ST307	NDM-1, NDM-5	3	3.57
ST580	NDM-1	3	3.57
ST1	KPC-2	2	2.38
ST14	NDM-1	2	2.38
ST278	NDM-1	2	2.38
ST20	NDM-1	1	1.19
ST36	NDM-1	1	1.19
ST147	NDM-5	1	1.19
ST273	NDM-1	1	1.19
ST281	NDM-1	1	1.19
ST322	NDM-1	1	1.19
ST617	NDM-5	1	1.19
ST831	NDM-5	1	1.19
ST1128	KPC-2	1	1.19
ST1466	NDM-1	1	1.19
ST1869	KPC-2	1	1.19
ST5502	NDM-5	1	1.19
untyped	NDM-1、IMP-4	3	3.57

## Discussion

KP is one of the most common gram-negative pathogens that causes nosocomial infections, especially in immunocompromised patients. With the widespread use of carbapenems antibiotics, the incidence of CRKP infections has increased. According to the China Antimicrobial Surveillance Network (https://www.chinets.com/Data/GermYear), the resistance rates of KP to imipenem and meropenem were 25.0% and 26.3% in 2018, and 22.6% and 24.2% in 2022, respectively. The resistance rates in China had slightly decreased over the past five years but remained at a high level. At present, the antibiotics that can be used for CRKP infections are limited, although colistin and tigecycline shown susceptibility in vitro, their usage in clinic were limited due to the toxicity and high economic burden [13]. The novel antibiotic ceftazidime-avibactam is recognized as treatment alternative, however, when it came to MBL-producing CRKP, it was ineffective [13]. As lack of effective antibiotics, explored the resistance mechanism and molecular epidemiology of CRKP may contribute to the management and control of the prevalence of CRKP infections.

The 103 CRKP strains in our study mainly came from ICU, traditional Chinese medicine department and neonatology department. Patients from ICU usually had multiple underlying diseases and were critically ill, and commonly performed invasive procedures, and exposed to carbapenems, which increased the risk of CRKP infections [5]. Patients from neonatology department may suffer from prematurity, low birth weight, intrauterine infection, low immunity, invasive procedures, exposed to carbapenems, which increased the risk of CRKP infections [14]. In addition, we also found that traditional Chinese medicine department had a high incidence of CRKP infection, maybe because of the admission of a considerable number of seriously ill patients with underlying diseases to the Chinese medicine department, which was a distinctive characteristic of our hospital. As a result, the presence of such patients contributed to the high incidence of CRKP infections.

Our study revealed that CRKP strains showed high resistance to most antibiotics, including carbapenems, cephalosporins, aztreonam, quinolones and β-lactam/β-lactamase inhibitor combination. Previous reports revealed that two major types of resistance mechanisms related to CRKP drug resistance. One is the expression of AmpC enzymes or extended-spectrum β-lactamases (ESBLs) combined with upregulation of efflux pump system or mutation of outer membrane proteins or alteration of penicillin-binding proteins, which make CRKP resistant to cephalosporins and monobactams [9, 15]. The other one is the expression of carbapenemases, which pose even more challenge, and cause CRKP resistant to almost all available β-lactams, including the carbapenems [2, 15].

In our study, among the 103 CRKP strains, carbapenemases genes were detected in 91 strains, and 68 strains (74.73%) produced MBL, indicated that the MBL was the main cause of KP resistance to carbapenems in our hospital, which was consistent with previous report in Chongqing, China [16]. However, it was different from the reports that KPC was the most prevalent enzyme in China [17–19]. Among the carbapenemases produced in 91 strains, NDM-1(41.76%) was found to be the most common gene, follow by KPC-2 (27.47%) and NDM-5 (27.47%). NDM-1 was first reported in a Swedish patient traveled to New Delhi in 2008 [20], and has been disseminated worldwide since then [21]. In China, NDM-1-producing Enterobacter was first reported in 2012 [22], and later mostly reported in children [23–26]. The CRKP strains in our study mainly came from neonatology department(13.59%, 14/103)and pediatric department (9.71%,10/103), which may made the NDM-1 to be the main mechanism leading to carbapenems resistance in our hospital. In addition, NDM-5 (27.47%) was also prevalent in our hospital. NDM-5 was first detected in a multidrug-resistant Escherichia coli ST648 isolate in the United Kingdom in 2011 from a patient with a recent history of hospitalization in India [27]. Since then, it has been spread worldwide [28–31]. Compared to NDM-1, NDM-5, with a 2-amino-acid mutation, shown elevated resistance to carbapenems and broad-spectrum cephalosporins[27], and the IncX3 plasmids mediated the horizontal transmission of bla_NDM−5_ gene [31–33].

In this study, we found that the major MLST typing of CRKP were ST11, follow by ST15 and ST17. ST11 is a single-locus variant (tonB) of ST258, and both them belong to the clone complex CC258 [15]. ST258 is prevalent in the United States and several European countries[34], while ST11 is the most common type in Asia, and accounts for up to 60% of CRKP in China [35, 36]. In addition, we found that NDM-1-producing CRKP ST17 isolates had an outbreak in our hospital, and the isolates mainly came from neonatology department. Newborns are vulnerable to CRKP infections due to their low immunity, and worse more, the choice of antibiotic therapy are relatively limited for them. To effectively control these CRKP infections, a comprehensive approach that combines strict infection control measures and antimicrobial stewardship is essential. Here are some key measures to consider: raising healthcare staff awareness through education and emphasizing the importance of proper hand hygiene before and after patient contact, implementing contact precautions for patients infected with CRKP, and ensuring appropriate disinfection of environmental surfaces and equipment [37]. In addition, strict antimicrobial stewardship is critical in reducing the emergence of antibiotic resistance, includes appropriate prescribing of antibiotics, and optimizing treatment duration and dosing.

## Conclusion

The CRKP isolates in our study shown resistance to a variety of antibiotics. Different resistance genes associated with antibiotics resistance in CRKP, and NDM was the predominant carbapenemase gene detected. ST11 was the major MLST typing of CRKP in our hospital. Since the high resistance to most antibiotics, it is extremely necessary to take measures to control the spread of CRKP infections, timely investigation of epidemiology should be performed, monitoring of antibiotic resistance mechanisms should be strengthened.

## Data Availability

The datasets analyzed during the current study are available from the corresponding author on reasonable request.
